# More Prehistoric Dentistry

**Published:** 1885-04

**Authors:** W. H. Waite

**Affiliations:** Liverpool, England


### MORE PREHISTORIC DENTISTRY.

BY W. H. WAITE, D. D. S., LIVERPOOL, ENGLAND.

There are in the "Brown Museum," in Liverpool, two specimens similar to those figured in the January number of the INDEPENDENT PRACTITIONER.

One consists of a gold band enclosing the right upper canine and left central, with spaces and rivets for right lateral and central. The artificial crowns are missing, but the two natural teeth are in good preservation.

The other is a similar gold band enclosing two laterals, in order to carry two artificial centrals. The latter are evidently carved out of some hard ivory, and make a fair substitute. They are in good condition, with the gold rivet passing right through, but the natural teeth to which this case was attached are wanting.

These precisely resemble the specimens illustrating Dr. Van Marter's paper, and do not need further illustration.

Judging from the fact that these gold bands, which are about an eighth of an inch deep, must have passed up well on to the necks of the natural teeth, and also consdering the depth of the artificial crowns, I am disposed to think these two cases were designed to replace teeth which had loosened and come away, rather than teeth which had decayed. It is difficult to conceive of these deep bands going up into place, if the gums and alveolar were in normal position.

The specimens I have seen belong to a very celebrated collection of antiquities made by Mr. Mayer, of this city, and presented by him to the museum in 1867. Beyond this I could learn nothing, except the fact that they are recorded to have been found in Etruscan graves.

